# Adipose–muscle crosstalk during the menopausal transition: mechanistic links to sarcopenic obesity in midlife women

**DOI:** 10.3389/fendo.2026.1805067

**Published:** 2026-05-04

**Authors:** Weixin Zhang, Qianhao Wu, Qianyuan Chen, Wenxing Qin, Dongfang Zhang, Qingrong Xu, Peipei Han, Ji Sun

**Affiliations:** 1 Jiangwan Hospital of Hongkou District, Shanghai University of Medicine and Health Sciences, Shanghai, China; 2 Collaborative Innovation Center for Biomedicines, Shanghai University of Medicine and Health Sciences, Shanghai, China; 3 Department of Medical Oncology, Fudan University Shanghai Cancer Center, Shanghai, China

**Keywords:** mechanism, menopausal transition, midlife women, rehabilitation, sarcopenic obesity

## Abstract

Midlife represents a biological “phenotype transition” for women, during which sarcopenic obesity risk can rise despite stable BMI. Evidence anchored to the STRAW + 10 framework suggests that the interval surrounding the final menstrual period is an acceleration window for fat gain and centripetal/ectopic redistribution, creating an “invisible remodeling” trajectory that may increase the risk of developing sarcopenic obesity, which is not captured by routine anthropometrics. We synthesize mechanisms linking menopausal biology to increased risk of sarcopenic obesity: obesity-driven inflammation, adipokine dysregulation, and lipotoxic flux that impair insulin–AKT signaling and metabolic flexibility, coupled with reduced contractile/endocrine muscle output that worsens adipose phenotype. A key mediator is myosteatosis, comprising intramyocellular lipid and intermuscular adipose tissue, which can reduce specific force and manifest as dynapenia even when muscle size is preserved. Scalable monitoring of muscle quality using ultrasound echo intensity may complement CT/MRI in midlife practice. Estrogen withdrawal may further amplify energetic and regenerative vulnerability via ERα-related mitochondrial quality control and satellite-cell support, although menopause-stage human longitudinal data remain limited. In the GLP-1 era, lean mass loss during weight reduction and rapid regain after withdrawal strengthen the case for a function-first strategy integrating progressive resistance training and per-meal protein dosing/distribution. Standardized, menopause-calibrated sarcopenic obesity definitions and cohorts integrating strength, muscle quality, and ectopic fat are key priorities for prevention.

## 1 Introduction

Sarcopenic obesity (SO) refers to a condition where there is excessive body fat and impaired skeletal muscle mass or function (1). Various criteria have been used to define SO in previous studies (**Table 1**). The consensus of the European Society for Clinical Nutrition and Metabolism (ESPEN) and the European Association for the Study of Obesity (EASO) defines SO as the presence of both obesity and sarcopenia (2). This phenotype is increasingly closely associated with adverse cardiovascular metabolic and functional outcomes (3). Especially during the middle-aged stage of women, as they experience a decrease in lean mass and an increase in fat mass, their body composition and metabolic risks may still undergo significant changes (4–7). It is worth noting that screening approaches based on an increase in body mass index (BMI) or an expansion of the waist circumference may not detect women in the middle-aged period who have a normal weight range but high fat content or poor fat distribution (6, 8). These phenotypes have a great correlation during the menopausal transition period (6, 9). We define middle-aged women as those between the late reproductive stage and the early menopause stage, using the STRAW + 10 framework for definition (late reproductive period, early/late menopausal transition period, and early menopause), typically corresponding to women in their mid-40s to mid-50s, and the time period before and after the final menstrual period (FMP) as the key turning point for body composition (10).

**Table 1:** Current research on sarcopenic obesity in middle-aged women.

Participants	Definition of SO	Study design	Body composition measurement methods	Key findings	Ref.
4,766 Korean females (42–52 years), median follow-up duration of 9.1 years	ASM index < 5.7 kg/m^2^ combined with PBF ≥ 35%	Longitudinal study	BIA	SO increases across the menopausal stages and becomes more pronounced from the late transition.	(20) 2025
Postmenopausal Thai women (n = 248; age 45–80 years)	Weight-adjusted skeletal muscle mass (SMM/W) < 35.6% with fat mass > 41%	comprehensive cross-sectional study	BIA	Nutritional factors and menopausal hormone therapy represent modifiable influences on SO development, which in turn is linked to a higher risk of osteoporosis in postmenopausal women.	(21) 2025
Postmenopausal Turkish women (n=300; 59.71 ± 9.9 years)	Muscle mass < 0.823 kg/(kg/m²), Handgrip strength < 22 kg, and Body fat > 60th percentile (population-specific threshold > 41%)	cross-sectional study	BIA	Among the patients evaluated, 15.4% had SO.	(22) 2025
Postmenopausal Korean women (n = 4,150;mean age: 62.41 years)	ASM index<23.0% and BMI≥25.0 kg/m^2^	cross-sectional study	DXA	Compared to simple obesity, SO has a greater impact on knee osteoarthritis.	(23) 2022

ASM, Appendicular skeletal muscle mass; BIA, Bioelectrical impedance analysis; BMI, Body mass index; DXA, Dual-energy X-ray absorptiometry; PBF, Percent body fat; SO, Sarcopenic obesity.

Midlife represents not merely a chronological midpoint but a biological “phenotype transition” for many women (4, 11). Longitudinal evidence indicates that the interval around the FMP constitutes an acceleration window for body composition remodeling, with fat mass increasing more rapidly and lean mass decreasing beginning roughly two years before the FMP (annual increase rate of fat mass: 1.7%; annual decrease rate of lean mass: 0.2%) and continuing into early postmenopause—patterns that are more clearly reflected than those of body weight or BMI trajectories (5, 6). In this context, relying on body weight or BMI alone can mask clinically meaningful repartitioning of fat and lean tissues—an “invisible remodeling” trajectory that may represent an early phase increasing the risk of SO earlier than traditionally appreciated (5, 12).

Crucially, early deterioration in midlife women may be functionally and qualitatively driven rather than solely quantified by muscle mass. Contemporary muscle health frameworks emphasize that functional impairment (e.g., strength) and tissue composition are not interchangeable with muscle quantity (13). Fat infiltration into skeletal muscle (myosteatosis) is increasingly recognized as an ectopic fat depot that correlates negatively with strength and mobility and is linked to metabolic dysfunction, including insulin resistance and diabetes (14, 15). Thus, the risk of SO in midlife women is plausibly shaped by a combination of increasing adiposity and early declines in muscle quality and performance—changes that may occur without dramatic losses in scale weight (1, 6).

Mechanistically, the menopausal transition provides a compelling biological backdrop for SO (4, 11). Beyond systemic fat redistribution, estrogen signaling supports skeletal muscle mitochondrial function and metabolic homeostasis, and reduced estrogen action can compromise these pathways (11, 16). Experimental and translational evidence further suggests that estrogen deficiency can impair the maintenance and regenerative capacity of satellite cells, providing a cellular rationale for reduced repair and recovery potential as estrogen declines (17). These changes intersect with a broader inflammatory–metabolic axis: inflamed adipose tissue promotes lipotoxicity and the release of pro-inflammatory mediators that impair insulin action, while declining muscle metabolic activity lowers resting energy expenditure and physical capacity, further facilitating fat gain—together establishing a self-reinforcing cycle (18, 19). In this mini-review, we therefore focus on SO as a midlife women’s health issue, highlighting how menopausal biology, muscle quality (including myosteatosis), and adipose–muscle crosstalk may jointly accelerate cardiometabolic risk, and we outline key gaps that limit prevention and intervention strategies.

## 2 Obesity-driven pathways that compromise skeletal muscle in midlife women: inflammation, anabolic sensitivity shifts, and myosteatosis

Midlife women frequently experience a phenotype shift in which body composition reorganizes, while total body weight or BMI do not accurately reflect these changes, with progressive visceral/ectopic fat expansion and concurrent deterioration in muscle performance. In this context, the development of SO is increasingly viewed not as a simple “sum” of fat gain plus muscle loss, but as a pathophysiologic synergy in which adiposity-derived signals accelerate muscle dysfunction and metabolic risk (5, 20, 24–26). Contemporary consensus frameworks provide an essential reference point for defining and staging SO, yet the midlife female phenotype exposes where population-level tools may under-capture early decline dominated by muscle quality and function (2).

### 2.1 Low-grade inflammation and adipokine imbalance as early drivers of muscle vulnerability

Obesity is characterized by chronic, low-grade inflammation that impairs skeletal muscle metabolic homeostasis (27). In midlife women, this inflammatory load is clinically relevant because adipose-derived endocrine signals can track functional vulnerability beyond BMI. Mechanistically, pro-inflammatory signaling and lipid oversupply may impair glucose disposal and promote ectopic lipid deposition, creating a milieu that may reduce the efficiency of anabolic remodeling and contribute to early declines in muscle quality (28). In parallel, adipokine dysregulation—particularly reduced adiponectin activity and increased leptin tone/leptin resistance—has been implicated in skeletal muscle insulin resistance, oxidative stress, and reduced metabolic flexibility in obesity-related settings, although direct menopause-stage evidence in midlife women remains limited (29, 30). Together, these processes may increase vulnerability to early muscle quality decline and ectopic fat infiltration before overt mass loss becomes apparent.

### 2.2 Anabolic responsiveness in midlife women: dose dependence, context dependence, and meal distribution

In obesity and metabolic dysfunction, “anabolic resistance” is used to describe an attenuated skeletal muscle protein synthetic response to anabolic stimuli such as dietary amino acids, insulin/IGF-1 signaling, and resistance exercise (31). Mechanistically, nutrient- and growth factor–dependent regulation of muscle protein synthesis converges on the PI3K–AKT–mTORC1 pathway, and obesity-related inflammation and lipid oversupply are consistently associated with impaired insulin signaling and reduced downstream activation of anabolic signaling nodes in skeletal muscle, which can limit the magnitude of postprandial and post-exercise remodeling (32–34). However, direct evidence in midlife women does not support a uniformly blunted anabolic response. Controlled feeding studies indicate that ingestion of an adequate high-quality protein bolus (e.g., ~25 g whey) can elicit a robust postprandial increase in myofibrillar protein synthesis in middle-aged women, suggesting that anabolic capacity may be preserved when the stimulus is sufficiently large and protein quality is high (35). Therefore, rather than describing midlife women as having an intrinsic anabolic resistance phenotype, the current evidence is better interpreted as supporting dose dependence and context dependence. A relative reduction in anabolic efficiency may be more plausible in subgroups such as women with obesity, metabolic dysfunction, or during energy restriction (34, 36, 37). This has practical implications for habitual dietary patterns, because protein intake is commonly skewed toward the evening meal (38); controlled comparisons of isonitrogenous diets demonstrate that a more even distribution pattern approximating ~30 g protein at breakfast, lunch, and dinner yields a higher 24-hour myofibrillar protein synthesis rate (≈25% higher) than a skewed pattern concentrating most protein at dinner (39). Collectively, these data support focusing the discussion in midlife women on modifiable determinants of anabolic outcomes—per-meal protein amount, protein quality (including essential amino acid/leucine content), and meal distribution—rather than presuming a uniform anabolic defect across this population (39–41).

### 2.3 Adipose–muscle crosstalk as a bidirectional amplifier: adipokines meet myokines

Adipose–muscle crosstalk relevant to SO is bidirectional in the literal sense that adipose tissue actively alters skeletal muscle metabolism and remodeling (adipose → muscle), and skeletal muscle—through contractile activity and secreted factors—also actively regulates adipose tissue phenotype and energy handling (muscle → adipose) (42). On the adipose → muscle side, obesity-related adipose expansion is accompanied by immune cell infiltration and endocrine dysregulation, characterized by higher circulating and local pro-inflammatory mediators (e.g., TNF-α, IL-6–linked signaling, MCP-1–related pathways), adipokine imbalance (relative reduction in adiponectin activity and increased leptin tone/leptin resistance), and increased delivery of lipid substrates and lipotoxic intermediates (e.g., elevated NEFA flux and bioactive lipids such as ceramides/diacylglycerols) (43, 44). These signals converge in skeletal muscle on impaired insulin signaling (reduced IRS-1/AKT pathway efficiency), mitochondrial overload and oxidative stress, and reduced metabolic flexibility, thereby promoting ectopic lipid accumulation within and around muscle and attenuating anabolic remodeling capacity (45, 46). On the muscle → adipose side, skeletal muscle functions as an endocrine organ whose secretome is strongly activity-dependent: contraction-associated myokines (e.g., IL-6 in its acute exercise-associated pattern) can influence adipose lipolysis and substrate utilization, IL-15 has been linked to regulation of adipose mass and oxidative phenotype, and myostatin is a negative regulator of muscle anabolism that is also associated with a more adverse body-fat profile (47, 48); irisin (FNDC5-derived) has been shown in experimental settings to promote thermogenic gene programs (including UCP1-related pathways), although the magnitude and consistency of this mechanism in humans remains variable across studies (49). When muscle mass, strength, and habitual contractile activity decline—as can occur during midlife weight gain and reduced activity—total substrate disposal and resting energy expenditure decrease, and the activity-dependent myokine profile shifts in a direction that is less supportive of adipose oxidative/thermogenic remodeling and more permissive of adipose expansion and inflammation (50). Taken together, “bidirectional” crosstalk in SO refers to two coupled processes: obesity-driven adipose endocrine/lipotoxic signaling that impairs muscle metabolism and function, and reduced muscle contractile/endocrine output that worsens adipose tissue phenotype, jointly amplifying fat accumulation, metabolic dysfunction, and functional decline (42).

### 2.4 Myosteatosis: IMCL vs IMAT, dynapenia, and scalable assessment using ultrasound

Beyond total muscle mass, muscle quality is strongly shaped by ectopic fat infiltration (14, 51). Myosteatosis includes at least two biologically distinct compartments. Intramyocellular lipids (IMCL) are lipid droplets within muscle fibers; they can reflect adaptive fuel storage in trained states but become maladaptive when coupled with mitochondrial stress, oxidative imbalance, and impaired lipid oxidation (52). Intermuscular adipose tissue (IMAT) refers to fat between muscle groups and fascial planes; it can act as a local inflammatory depot, promoting paracrine immune signaling and impairing contractile performance (53).

Myosteatosis provides a biologically plausible explanation for “hidden” deterioration in BMI-stable midlife women: even when muscle cross-sectional area appears preserved, fat infiltration may be accompanied by deterioration in muscle quality, suggesting early tissue-level decline that may precede overt strength loss (54). Much of the strongest imaging–function evidence linking lower muscle density/attenuation to reduced strength comes from older cohorts, supporting biological plausibility that infiltration depresses functional output beyond what muscle size predicts; nevertheless, this should be treated as mechanistic anchoring rather than direct midlife-specific effect estimation (55).

In the past, ultrasound, bioimpedance analysis (BIA), CT, MRI, and dual-energy X-ray absorptiometry (DXA) have all been used clinically to assess muscle quality (56). To strengthen clinical relevance for midlife practice, muscle quality assessment should not rely exclusively on CT/MRI. Ultrasound echo intensity (EI) is a feasible, scalable surrogate for muscle quality that can support repeated monitoring across the menopausal transition in settings where advanced imaging is impractical. Emerging longitudinal work in women across menopausal stages suggests that muscle size can remain relatively stable while EI increases, consistent with progressive intramuscular fat/connective tissue infiltration and supporting the “invisible remodeling” concept (54). Because EI is influenced by device settings and subcutaneous fat thickness, longitudinal applications should emphasize standardized acquisition and, where feasible, corrected EI (or clearly specified normalization procedures) to improve comparability across time and individuals (57). In addition, DXA offers the advantages of high accuracy, wide availability, and low radiation exposure (56). It is widely reported to be more acceptable and comfortable for patients, and it can provide multiple body composition indices within minutes (58). In DXA, X-rays pass through the human body at two different energy levels and undergo attenuation (58, 59). Depending on the intensity of the emitted energy, as well as the density and thickness of anatomical structures and tissues, the radiation energy is attenuated to varying degrees (58). This allows for the analysis of muscle quality. In a study by Greendale et al. (5) on changes in body composition and weight during the menopause transition, DXA provided accurate measurements of fat mass and lean body mass. The study revealed that an accelerated increase in fat mass and a decrease in lean body mass are phenomena associated with the menopause transition (5).

## 3 The menopausal transition as an amplifier: estrogen loss, mitochondrial stress, and impaired regeneration

### 3.1 ERα and mitochondrial quality control: preclinical mechanisms and translational relevance

Skeletal muscle estrogen receptor signaling, particularly via ERα, is implicated in maintaining mitochondrial function and metabolic homeostasis (60). Strong causal support for ERα-related mitochondrial mechanisms comes primarily from preclinical models, including skeletal muscle–specific ERα disruption, which is associated with impaired mitochondrial metabolism and reduced metabolic flexibility (11). These data provide mechanistic plausibility for menopause-associated energetic vulnerability but do not, by themselves, establish that identical fission–fusion or mitophagy defects occur in midlife women (61).

Mitochondrial quality control is best conceptualized as a coordinated system involving mitochondrial dynamics (fission/fusion) that segregate damaged components and preserve network function, and mitophagy that clears dysfunctional organelles. In a midlife SO context, these control nodes plausibly intersect with lipid overload: if mitochondrial remodeling and clearance are less efficient during estrogen withdrawal, lipid oxidation may lag behind lipid delivery, predisposing to IMCL accumulation, oxidative stress, and reduced metabolic flexibility (62). Direct menopause-stage–anchored biopsy studies in midlife women that quantify these pathways remain limited, defining a key translational gap (63).

### 3.2 The ROS–FoxO–atrogene axis

A key downstream consequence of impaired mitochondrial maintenance is increased oxidative stress. Elevated ROS can shift muscle toward catabolism by enabling transcriptional programs that upregulate ubiquitin–proteasome and autophagy-related degradation (64, 65). Canonically, reduced PI3K/AKT tone permits FoxO activation, which induces atrophy-related ubiquitin ligases such as atrogin-1/MAFbx and MuRF1 (66, 67). This axis provides a mechanistic bridge between energetic stress and functional decline, and it underscores how obesity-linked metabolic stressors may synergize with menopause-linked shifts in cellular homeostasis (68).

### 3.3 Impaired regeneration: satellite cell biology and transcriptomic signals

The menopausal transition may influence skeletal muscle regenerative capacity through effects on the satellite cell compartment, which is required for myofiber repair and remodeling after injury, disuse, and training (69). The strongest causal evidence comes from preclinical models: estradiol signaling acting through ERα in satellite cells maintains satellite cell number by limiting apoptosis, and loss of estrogen–ERα signaling reduces satellite cell content and impairs strength recovery following muscle injury (70). Human evidence is more limited but directionally consistent: biopsy observations in women transitioning from peri- to postmenopause have reported reductions in satellite cell indices coincident with declining estradiol (17), and a double-blind randomized trial in early postmenopausal women undergoing resistance training found that satellite cell measures declined in the placebo group but were counteracted when resistance training was combined with transdermal estrogen therapy (with divergent changes in satellite cells per fiber across fiber types) (71). These data support the interpretation that estrogen status can modulate satellite-cell–related remodeling *in vivo*, but they also highlight key constraints for midlife inference: satellite-cell readouts in human studies can be influenced by participant age, training status, exercise mode, and follow-up time after exercise; therefore, comparisons across studies and menopause-stage sampling protocols require careful standardization (72). Complementing these cellular measures, longitudinal transcriptomic studies across the menopausal transition have begun to identify coordinated shifts in gene programs relevant to tissue maintenance and repair (e.g., cell-cycle control, extracellular matrix remodeling, and myogenesis-related signaling), but these signatures should be interpreted as molecular associations rather than direct evidence of altered satellite cell kinetics. Collectively, the current evidence supports a biologically plausible menopause-linked reduction in regenerative support, while underscoring the need for menopause-stage–anchored longitudinal studies in midlife women that jointly quantify satellite cell indices, muscle quality, and strength outcomes under standardized sampling conditions (64).

## 4 Rehabilitation and intervention strategies: breaking the adipose–muscle vicious cycle in midlife women

### 4.1 Exercise as the cornerstone: metabolic unloading and strength preservation

In obese midlife women, exercise should be framed not only as “calorie expenditure,” but as a targeted strategy to attenuate obesity-driven inflammatory signaling, restore metabolic flexibility, and protect cardiac function (73–76). Aerobic exercise can be positioned as metabolic unloading—reducing ectopic lipid burden, improving insulin sensitivity, and lowering systemic inflammation—while progressive resistance training (RT) directly targets dynapenia and supports muscle quality (77, 78). Because obesity-related inflammation and lipotoxic stress can blunt the normal rise in muscle protein synthesis after amino acids and exercise, RT prescriptions should be progressive, adequately loaded, and sustained, with individualized safety considerations including symptom burden, joint health, bone health risk, and training history (79, 80).

### 4.2 Nutrition to overcome anabolic sensitivity shifts: per-meal thresholds and targeted distribution

Because anabolic responsiveness in midlife women appears to be context-dependent rather than uniformly impaired, and may be less efficient in subgroups such as women with obesity, metabolic dysfunction, or during energy restriction, nutrition should be framed as a precision strategy to repeatedly deliver a meaningful anabolic stimulus rather than a generic healthy-eating add-on (31, 34, 37). Practically, an evidence-informed scaffold is to ensure adequate total daily protein when the goal is lean-tissue preservation during weight management or functional decline, with explicit individualization to renal function, comorbidity burden, and energy sufficiency (81–83). Within this context, distribution becomes mechanistically relevant because muscle protein synthesis is saturable per eating occasion; therefore, recommendations should move beyond “even spacing” toward per-meal boluses that reliably reach an essential amino acid/leucine trigger, particularly in sensitivity-reduced states where sub-threshold meals may fail to stimulate synthesis meaningfully (84). Importantly, this practical emphasis on adequate per-meal dosing does not imply that midlife women are uniformly “resistant” (37); rather, it acknowledges that robust responses are often preserved when the stimulus is sufficient, while everyday meal patterns frequently do not reach the trigger required to generate repeated daily anabolic pulses (85). These dietary tactics are most defensible when paired with progressive RT, because combined mechanical and amino-acid signaling offers the most plausible route to preserve strength and muscle quality during the midlife transition (86, 87).

### 4.3 Weight-loss interventions in the GLP-1 era: implications for muscle preservation

Substantial weight loss—whether achieved pharmacologically, surgically, or through caloric restriction—typically reduces both fat mass and lean mass, and the composition of weight loss is clinically relevant when the goal is to prevent sarcopenic obesity in midlife women. Recent evidence from GLP-1 receptor agonists and GLP-1/GIP co-agonists indicates that lean mass decreases alongside fat mass during treatment, highlighting that scale-based success does not necessarily equate to preservation of muscle-related reserve (88, 89). In addition, randomized withdrawal studies show that discontinuation is commonly followed by substantial weight regain and partial reversal of cardiometabolic benefit (90, 91). In midlife women, this pattern is particularly relevant because menopause-related shifts in fat distribution and muscle vulnerability may make long-term outcomes more dependent on preservation of strength and muscle quality than on body weight alone. Therefore, GLP-1-based weight management should be implemented within a function-first framework, in which progressive resistance training, adequate protein intake, and monitoring of strength and body composition are treated as core co-therapies rather than optional adjuncts (88–91).

## 5 Conclusion

In midlife women, the development of SO is best conceptualized as arising from an invisible remodeling trajectory in which menopause-linked centripetal fat redistribution and ectopic deposition can progress even when changes in body weight or BMI do not fully reflect the underlying body composition remodeling, uncoupling routine anthropometric measures from true metabolic and functional risk. While consensus definitions are essential for standardization, midlife women expose a remaining gap: early decline may be dominated by deteriorating muscle quality and strength and may therefore be under-recognized by BMI/WC-anchored screening in the absence of menopause-calibrated thresholds. Mechanistically, obesity-related inflammation and context-dependent shifts in anabolic sensitivity intersect with estrogen-withdrawal–linked energetic and regenerative vulnerabilities supported strongly by preclinical and translational evidence but incompletely mapped in menopause-stage–anchored human cohorts. In the GLP-1 era, the long-term risk is shaped less by drug-specific muscle harm than by weight cycling after withdrawal and potential asymmetric regain, underscoring a function-first paradigm that tracks strength and muscle quality alongside adiposity and prioritizes resistance training plus per-meal protein strategies that reliably reach an essential amino acid/leucine trigger, individualized to comorbidity and feasibility, during this last major window before later-life disability trajectories consolidate.
